# Life Themes and Attachment System in the Narrative Self-Construction: Direct and Indirect Indicators

**DOI:** 10.3389/fpsyg.2019.01393

**Published:** 2019-06-18

**Authors:** Giulia Di Fini, Fabio Veglia

**Affiliations:** Department of Psychology, University of Turin, Turin, Italy

**Keywords:** narration, Life Themes, attachment, internal working models, meaning making

## Abstract

The narration is the instrument that allows individual to organize his experience through a process of attribution and sharing of meaning within social interactions and to translate the extraordinary in normativity. As regards the narrative content there are “narrative genres” that impose thematic constraints on the way of narrating, but these also allow creative variations. According to the theoretical-clinical hypothesis of Life Themes (LTs), Love, Value, Power, Freedom, and Truth are shared attractors of meaning recurring in Life Stories. The influence of attachment system on the development of autobiographical narratives is shown in literature. This article presents a qualitative study of the specific dimensions and valences of the LTs in the adult attachment narratives. The study is part of a broader research project that aims to systematize the theoretical model of LTs in the narrative Self-construction, to study its connections and applications in the field of attachment theory, and to build a useful operative tool in the clinical assessment of patients’ stories. We also aimed to study the occurrence of the LTs in the Adult Attachment Interview transcripts, preliminarily exploring possible connections with Internal Working Models. 40 AAI transcripts of 20 men and 20 women (mean age was 33 years old), selected from a larger non-clinical sample employed in an adult attachment study, were analyzed using a phenomenological exploratory approach. For the research design we used a qualitative methodology that ensure a deep and intensive, rather than extensive, analysis, focusing on the complexity of meanings and discussing the results within the research group. Several indicators of LTs have been identified and the interpersonal dimensions were explored. An additional Theme emerged from the recurrence of some indicators: Ethic and Justice. In Dismissing subjects’ narrations the theme Value (in terms of being valued) emerges mostly, while in the Preoccupied ones the theme Power (in terms of impositions received), accompanied secondarily by Value and Freedom, occurs more frequently. The most frequent thematic indicators in the F group are related to Love and Truth. Clinical implications of an explicative model of critical Themes are discussed.

## Introduction

The narrative approach in clinical psychology (Bruner, 1990, 1997, 2003) focuses on the biological need of purpose and meaning that drives human beings to the use of narration as a mean of reorganization of one’s own stream of experiences, making it understandable for oneself and sharable with others (Spira and Wall, 2006). Human beings are peculiar in their ability to arbitrarily select (Damasio, 1999, 2010) and interpret events (Gazzaniga, 2012) and shared narrative themes are central for integrating perceptions of roles and relations (Singer and Bonalume, 2008). The theoretical-clinical hypothesis of Life Themes (LTs) postulates the existence of powerful attractors of meanings, beyond individual and cultural variations (Csikszentmihalyi and Beattie, 1979; Veglia, 2013; Veglia and Di Fini, 2017). LTs are fundamental epistemic motivational systems, driving and supporting the human brain’s endeavor to organize the interpretation of experiences by means of narration, ultimately generating countless unique stories, essential in developing self-concept and cultural affiliation. In literature some authors consider the existence of “nuclear” and constant themes that permeate life stories (McAdams, 2001), as well as constraints and opportunities for the development of the narrative self construction (Veglia, 1999, 2013). The identification of some main narrative themes started from the analysis of hundreds of clinical stories, the analysis of mythology, narrative literature, anthropological, philosophical, psychological, and neuroscientific essays, and a broad brainstorming carried out over several years by a research group within the Department of Psychology of the University of Turin and a clinical group within the Crocetta Clinical Center in Turin, both led by Fabio Veglia. Through this work some main criteria were considered for the formulation if the theoretical–clinical hypothesis of LTs. It was considered: the theme’s irreducibility; the utmost independence of each theme from contextual characteristics; the presence of the theme in every culture and every historical period examined; the intrinsic structural capacity of the theme to attract and generate meaning both in the form of expressions and in the construction of the self and of the dialogue between parts of the self (Veglia and Di Fini, 2017).

This hypothesis suggests the existence of a number of basic recurrent themes, common in any given personal narration and cross-cutting individual and cultural differences throughout history: Love, Value, Power, Justice, Freedom, and Truth (Veglia and Di Fini, 2017). Each theme seems to have intrinsic development lines that influence the self-perception, behaviors, cognitive and relational styles. The theme of Love, in direct continuity with the caregiving system, can be narrated as the ability of loving, being loved and being lovable. The theme of Personal Value includes the narratives related to personal characteristics that may be assigned and recognized within interpersonal relationships according to different criteria. The theme of Power is the narrative framework within which stories related to the system of social-ranking can be narrated; it also includes the ability to control self, the others, and the events, accompanied to the individual’s awareness of being active actor and author of a project. The theme of Freedom is tied to the concept of limits and boundaries needed to stem the experiences of anguish, confusion and disorientation that would result from its infinite extension. The theme of Truth is connected to the construction of meaning by the individual along his development. The theme of Justice includes narratives about moral and ethical principles that permeate the interpersonal relationships (Veglia and Di Fini, 2017).

In line with McAdams’s studies (McAdams, 2001; McAdams and Guo, 2015), death is accounted for as the necessary framing device in which every possible theme is developed, rather than a theme itself. Such definition is included in the concept of generativity: a creative act, a form of continuation of lineage and self-expression (Sugarman, 2001). The LTs can be thought using three polarized axes that describe a continuum of possible positions between different extremes (Veglia and Di Fini, 2017): Basic Control vs. Co-regulation vs. Sharing of the Theme, Absent/Negative Narration vs. Present/Positive Narration, Narrative Low Integration vs. Narrative High Integration.

Clinically, the inability to develop one or more LTs, by means of personal narrations, necessarily leads to a protracted lack of purpose and meaning: a profound distress builds up over time, eventually resulting in clinically relevant typical and atypical symptoms (Frankl, 1985; Kleftaras and Psarra, 2012; Siegel, 2012, 2014). Themes etiologically relevant are referred as critical narrative themes. Many different modalities (or variations of the themes) are used for writing one’s own history and promoting dialogue between the different parts of the personality (Guidano, 1988; Van der Hart et al., 2006; Knight and Falstein, 2014), enabling their flexible regeneration and rewriting (Veglia, 1999; Veglia and Di Fini, 2017). From a clinical point of view, the collection of salient information about both the subject’s narrative methods and the significant thematic contents, might provide the clinician with a deepened understanding of the emotional imbalance, connected to the development of a psychopathology.

From a cognitive-evolutionary point of view (MacLean, 1990), semantic initiators are thought to be continuous and interdependent from the Interpersonal Motivational Systems (IMS) and their representations (Liotti, 2001). Particular emphasis was put on the attachment system (Bowlby, 1969, 1973, 1980, 1982), due to its early contribution in establishing auto-referential nuclear meanings, that in turn generate a specific sense of Self (Guidano, 1988; Siegel, 1999, 2012, 2014).

Studies of the processes underlying narrative coherence, as well as structured thematic contents with regards to Internal Working Models (IWM), focus on the narrations of past attachment experiences. The Adult Attachment Interview (AAI – George et al., 1985), used clinically during the assessment and diagnostic phases, is the preferred instrument for investigating Self-representations and attachment relationships recounted autobiographically (Steele and Steele, 2008). Standard coding for AAI requires the qualitative analysis of transcripts, mainly focusing on the narration’s formality and coherence, rather than thematic content. Moreover, the thematic content analysis might provide the clinician with additional information for correctly monitoring the patient’s progresses, not only in terms of narrative coherence and reflective ability, but also as a measure of balanced development between critical themes.

The present study aims to investigate the occurrence of LTs in the transcripts of AAI. The primary objective regards the design of the operational definitions of the LTs, starting from the subjects’ own point of view and measuring their occurrence in the transcripts of AAI, thus in relation to past attachment experiences. The secondary objective regards a preliminary exploration of the possible connections between LTs and States of Mind in regard to attachment, according to the AAI’s standard coding.

The two phases of the present study aim to achieve the following specific objectives:

(1) Detecting the explicit and direct indicators of LTs by investigating what semantic nuances the participants attribute to them and which keywords they choose to describe them. The explicit indicators were measured through the added question to AAI protocol.
(2) Detect the implicit and indirect indicators of the LTs that emerge in those portions of transcripts in which the participants are asked to narrating past experiences of attachment. Indirect implicit indicators were investigated through the narration of past attachment experiences, obtained from the standard AAI interview.

From a clinical point of view, each goal is oriented to the construction of more effective instruments for devising caring plans, while at the same time providing a validation/reformulation of models of intervention.

## Materials and Methods

### Textual Corpus

Textual corpus consisted of 40 AAI transcripts randomly selected from a larger textual corpus of a hundred of AAIs collected in non-clinical convenience samples that were recruited for previous adult attachment studies (Di Fini, 2014 - unpublished doctoral dissertation). This study only used secondary data sources, composed by written texts (AAI transcripts). Thus it did not require further ethics approval or consent to participate. All participants in the original study gave written informed consent in accordance with the Declaration of Helsinki. The protocol of the original study was approved by the Bio-ethics Committee of the University of Turin. Each AAI was conducted face-to-face by researchers trained by Jacobvitz and Dazzi to administer the AAI. The interviews were recorded, transcribed verbatim and classified according to Main et al. (2003) coding system.

The transcripts were selected from the original samples in order to create a stratified by gender (50% M and 50% F) textual corpus; subsequently, each transcript was randomly selected in order to ensure compliance with the European Distribution of subjects (Bakermans-Kranenburg and van IJzendoorn, 2009), for the three-way (30% Ds, 56% F, and 14% E) and four-way (25% Ds, 52% F, 11% E, and 12% U) AAI classification. Mean age of adults was 33 years old (*M* = 32,76; *SD* = 7,33; min = 21, max = 58), 45% of the participants (*n* = 18) held a junior high school degree, 55% (*n* = 22) a university degree.

### Instruments

#### Adult Attachment Interview (AAI – George et al., 1985)

Adult Attachment Interview (AAI) is a semi-structured interview consisting of twenty questions about past attachment experiences, used in adults for evaluating the current state of mind toward such experiences. The interview requires the narration of specific events, in order to elucidate the quality of caring; subjects are asked to briefly outline the relationship with each care-giver, using adjectives, words or sentences. The narrative style and the grade of consistency between recalled events and parent’s descriptions are used to assign different “States of Mind.” AAI accounts for four states of mind: free-autonomous (F), entangled-preoccupied (E), dismissing (Ds), and unresolved (U). A random sample of AAI transcripts (25%) was independently coded by a second certified coder; both coders had been trained by Jacobvitz and Dazzi. Inter-rater agreement for the three-way classification (F, E, and Ds) was 90% (κ = 0.81, *p* < 0.01) and 86% (κ = 0.76, *p* < 0.01) for the four-way classification (F, E, Ds, and U). Coders resolved possible mismatches by means of discussion.

This peculiar interview provides a narrative report of some relational experiences, namely those associated with attachment. Such stories and many others, including friendship, love and belonging ones, constitute our own stories, all the more “true and embodied” (Veglia, 1999). Transcripts can be used for looking into themes which might have been associated with the life path, organizing the succession of narrative strings, imprinting a living, significant, meaningful stamp, detectable through even mere words.

A generative question was added to the AAI protocol in light of the present research, designed for directing the report, at the semantic level, toward the description of LTs, leaving the subject free to encompass mental associations, images, sensations, and emotions. Subsequently, the item is articulated in more detailed questions, aimed at a deeper investigation of the subject’s more relevant Theme and a related childhood experience, in line with the protocol style.

The item, called Question 21, is the following:

21. Now I am going to tell you five words, please describe which emotions are evoked by each of them. The words are: “love,” “value,” “power,” “freedom,” and “truth”.

Which word evoked the most intense emotion?

Which word evoked the less intense emotion?

Could you recall a childhood event connected to the first word and the emotion you just told me about?

Data gathering in relation to the LTs was possible through such questions, directing the subjects toward a more specific, but still well blended throughout the narration, reflection on the Themes. Coding for Question 21 was performed according to the subject’s own linguistic style during the whole interview and the heuristic shift between different memory registers, in order to avoid methodological inconsistencies that might have negatively impacted the natural flowing of narrations.

### Procedure

Qualitative Research guidelines (Smith, 2008) were followed to ensure a deep and intensive, rather than extensive, analysis, focusing on the complexity of meanings.

The AAI and the answers given to the added question no. 21 had been previously transcribed ad verbatim. The transcription process was made using Main et al. (2003) rules that include instructions to transcribe both verbal and non-verbal contents. The transcripts didn’t return to participants for comments or correction.

The first aim was to operationalize the LTs. We used an inductive approach through the Thematic analysis (Braun and Clarke, 2006): indicators were measured starting from the participant’s own point of view. Transcripts were analyzed using a phenomenological exploratory approach and assigned to a LT, resulting in five groups of texts. Textual analysis was conducted iteratively, progressively adding the answers, used gradually for bringing out the different dimensions of each Theme. Such cyclic process ended once “theoretical saturation” had been reached, indicating that no more details could be extracted from the texts. The point of saturation was determined by consensus of the research group (Marrow, 2005).

The second aim was measuring the occurrence of LTs in the answers given to the standard AAI protocol (20 items) and detecting the indirect indicators. We used a deductive approach in line with Content Analysis (Pope et al., 2000). During a first phase, textual segmentation was employed to identify textual fragments from which a thematic meaning could be inferred. Classification of every fragment was carried on starting from macro-Themes first and from the list of codes devised in the first aim second. Every textual unit could be assigned to more than one category. Hierarchical descending analysis was used to examine the categorized fragments, in order to identify an increasingly refined and detailed specification of the starting themes, namely promoting a partitioning of the initial categories into subcategories, able to accurately reflect the meaning of the texts.

As regards the procedures through the use of the software ATLAS.ti, an analysis unit (hermeneutic unit in ATLAS.ti) was open for every objective. Answers to Question no. 21 were grouped in sections for each Theme, resulting in a corpus testi of five transcripts (a hermeneutic unit was open in ATLAS.ti – HU1). Salient words (core-words) connected to one or more Themes were extrapolated by examining the context in which they were used, in order to later guide the scorer in evaluating direct indicators of LTs. They constituted the Thesaurus of each Theme.

Extensive reading of the transcripts lead to the identification of 380 textual fragments (quotations in ATLAS.ti), which were later assigned one or more labels (codes in ATLAS.ti). Codification resulted in the creation of 129 codes, later grouped by the two coders in macro-categories (Code Families in ATLAS.ti), through a conceptual process: they incorporate information contained inside the codes to a higher level of abstraction, constituting theoretical dimensions which encompass all the information provided by the empirical indicators. Therefore, the creation of Code Families by the two independent coders allows for the distinction and grouping of different indicators, in this case, semantically alike.

The investigation of narrative indicators for each semantic area allowed for the observation of “inter-Theme” features. We examined potential linkages between Themes and their own groups, through selective coding provided by the Co-Occurrence Analysis function, which calculates the number of common quotations between Code Families and the co-occurrence between codes.

Once coding of the answers no. 21 was performed, the complete AAI transcripts were analyzed using previously identified Code Families, as well as new ones subsequently integrated in the categorization system (HU2). Iterative analysis included a continuous matching between codes and Code Families. 978 quotations were coded (one or more labels could be assigned to each text fragment), resulting in a total of 70 codes. Code grouping resulted in 26 Code Families. In this phase, empirical indicators pertain interpersonal relationships with the caregivers, rather than abstract thematic suggested by the use of common terms and highly informative words about each Theme. Furthermore, the software made possible to qualitatively analyze codes and Code Families occurrence.

The whole coding process was carried out on the transcripts, after the interviews.

Furthermore, starting from the results obtained in this last phase, we observed the differences between the AAI classifications, qualitatively inferring possible links between Themes/state-of-mind with respects to attachment.

### Data Analysis

ATLAS.ti 6.0 (Atlas.ti Scientific Software Development, Berlin, Germany), a qualitative textual analysis software, was employed both for identifying the main themes emerging from the texts and comparing coding methods for the different coders. This software was designed for systematical identification and categorization of recurrent topics.

Two independent coders coded the transcripts. Transcript coding was followed by cross-matching both lists of codes and Code Families, via inter-rater reliability measured by Cohen’s kappa (*κ* = 80%). Possible mismatches were discussed between the judges until a sufficient level of agreement was reached.

Occurrence of codes and Code Families in both hermeneutic units was measured. Since more than one code could be assigned to each textual fragment, we were able to assess the correlation between coded elements using Boolean and semantic operators which explore the analytic categories (Query Tool function).

Kruskal–Wallis test in SPSS 18 was used to evaluate any significant statistical differences between the AAI groups, starting from the observed thematic categories.

## Results

### Designing Operational Definitions and Analyzing Direct Indicators

The first goal of the present study was detecting the shades of meaning attached to each Life Theme suggested in Question 21.

Grouping of emergent codes resulted in a total of 21 Code Families: through a conceptual process, 6 Families were created ad-hoc by the two coders, who discussed until a sufficient level of agreement was reached. These categories represent the shades of meaning attributed to the LTs by the subjects (Table 1); 2 Families for the negative and positive meaning attributed to the Themes (Table 2); 13 Families for constructs available in literature linked to the created codes (Table 3). Within the Families related to semantic dimensions, those including the largest portions of text are: Dimensions of Love (84 quotations) and Dimensions of Truth (53 quotations). Themes are more frequently interpreted as positive (130 vs. 40 quotations).

**Table 1:** Code Families in the first Hermeneutic Unit (HU1): *ad hoc* Code Families with examples from transcripts.

Code Families and codes	Codes	Quot.	Examples of quotations
		
	*n*	*n*	
Dimensions of Love [Love as physical attraction] [love as family value] [love as experience yet to be understood] [love as a life experience] [love as an all-encompassing experience] [love as trust] [love as passion, interest in something] [love as received material things] [conjugal love] [dedicated love] [exclusive love] [physical love] [incarnate love] [unconditional love] [love related to suffering] [mother’s love] [love in friendship] [love not received] [mutual love] [received love]	20	84	[1:73] “Surely all the moments shared in the family, Christmas, birthdays, all these moments, perhaps when you are young you pay attention to all these, these little attentions, these moments of warmth.” [1:40] “Love evokes trust and be trusted.” [1:77] “When I came home, and I always get what I asked for, a new toy, candy. For me it was the best thing at the time, that’s definitely love.”
Dimensions of Truth [Lack of truth] [hidden truth] [partiality of truth] [truths connected to freedom and to the value] [truth as accessory] [truth as the basis of relations] [truth as clarity] [truth as a condition of life] [truth as knowledge] [truth as dialogue] [truth as confidence] [truth as a guide] [truth as a relationship] [truth as respect] [truth as discovery] [truth as value] [truth as life] [truth to be transmitted] [objective/subjective truth]	19	53	[5:42] “It is also important to tell the truth to discuss calmly.” [5:46] “In the past all these truths are missing… this lack of clarity that I now strongly seek instead. I must know the truth, you have to explain it to me, do not leave any gray areas.”
Dimensions of Value [Mutability of values] [devaluation of value] [value as appreciation] [value as sharing] [value as courage] [value as a culture] [value as force] [value as pride] [value as power] [value as liability] [value as respect] [value as stability] [value as criterion] [value to be transmitted] [family value] [value of life] [value of things] [value as altruism] [value as love] [value as faith] [moral value]	21	43	[4:13] “Pride, a sense of pride.” [4:61] “Value is something that you have to conquer.” [4:60] “My value gives me security today. So it’s something that I have taken in the past and that can make me feel good, it gives me stability.”
Dimensions of Power [Management of power] [power as self-affirmation] [power as autonomy] [power as authority] [power as capacity] [power as discernment] [power as strength] [power as justice] [power as fight] [power as threatening] [power as implementation] [power as recognition] [power as will] [decision-making] [power at work] [power as abuse] [power as love] [power related to money] [power as danger] [power as possibility] [power as potential]	21	43	[3:48] “You should always manage power wisely, be very aware of what you have, so I would say reflection, awareness.” [3:54] “If you have power over someone, you can automatically manipulate him.”
Dimensions of Freedom [Independence] [freedom as self-discipline] [freedom as a condition for being loved] [freedom as a natural condition] [freedom as a conquest] [freedom as illusion] [freedom as discovery and opening] [freedom as value] [freedom of thought] [freedom of choice] [unconditional freedom] [inner freedom] [freedom limited by boundaries] [freedom of action] [freedom received]	15	41	[2:59] “Freedom does not exist because at the end you always have to compromise in every field and so it is a utopia, something unattainable.” [2:54] “Paradoxically, I can think of self-discipline.” [2:61] “When my mom lets me choose my own so I do not… I never felt forced.”
Ethics and Justice [Values as respect] [truth as guide] [power as justice] [value as liability] [value to be transmitted] [truth as respect] [loss of values]	**7**	**22**	[4:1] “The main value that comes to my mind is respect because it is a matter of life that I still have. Respect for people, for what they think and say. Do not try to argue too much if you have a different opinion, leave it without trying to change it.”


**Table 2:** Code Families in the first Hermeneutic Unit (HU1): positive and negative descriptions of Themes.

Code Families	Codes	Quot.	Codes
		
	*n*	*n*	
Positive descriptions	32	130	[Love as physical attraction] [love as the value of the family] [love as trust] [love dedicated] [unconditional love] [mutual love] [love received] [freedom as a condition for being loved] [freedom limited by boundaries] [freedom received] [lack of truth] [multiplicity of power] [love multiplicity] [mutability of values] [power as autonomy] [power as discernment] [power as recognition] [power understood as love] [recognition and appreciation of the value] [value as appreciation] [value as sharing] [value as liability] [value as respect] [value to be transmitted] [family value] [value intended as altruism] [value understood as love] [truth as the basis of relations] [truth as dialogue] [truth as confidence] [truth as a relationship] [truth as respect] [truth to be transmitted]
Negative descriptions	14	40	[Love as receiving material things] [love related to suffering] [love not received] [freedom as illusion] [freedom limited by boundaries] [lack of truth] [hidden truth] [partial truth] [loss of values] [power as threatening] [power understood as an abuse] [power understood as a danger] [devaluation of values] [value as loss of respect]


**Table 3:** Indicators about “Ethics and Justice” code Family.

Codes	Quotations
Truth as respect	[4:1] “The main value that comes to mind is respect because it is a matter of life that I still have. Respect for people, for what they think and say. Do not try to argue too much if you have a different opinion, leave it without trying to change it.”
Truth as a guide	[5:11] “I’m thinking about the future with my son, there are definitely many truths and nuances that change and you must choose the most right and accurate way.”
Truth to be transmitted	[4:56] “I can think of some notions that parents transmit to you and that you will carry with you. I have not had many values because my parents did not have… But in my conscience I realize what it is right and what is wrong and I hope to teach this to my daughter.”
Loss of values	[4:59] “The values have been lost, many moral principles are lost today.”
Power as justice	[3:29] “As a child I thought I had the power to fix some injustices maybe, like a super hero.”
Value as a responsibility	[4:61] “I associate a more concrete sense of responsibility to the value, something you have to conquer.”


Co-occurrence analysis underlines the association of peculiar pairs such as Freedom/Power, Value/Love, Love/Freedom, Freedom/Truth and Value/Power, thus indicating lines of development particularly linked. The theme Power as duty is linked to the theme Freedom as achieved independence. The theme Love appears as an ideal in its interpretation of non-conditioning, thus it is linked to both self-sufficiency and the ability to fulfill one’s own potential. A connection emerged between recognized personal Value, unconditional Love, the ability of being oneself (Power) and the Freedom of thought and expression.

The theme Truth is linked to the theme Freedom when the relationship is characterized by the presence (or absence) of freedom of expression, defined as the ability to express oneself without preoccupation toward any consequences or reactions. The theme Value is indirectly observed in those narrations where interpersonal relations are mediated by an underlying gap, an asymmetry of Power: the ability to develop a respectful and obedient attitude leads to the attribution of personal value.

When asked to narrate a childhood event, the majority of the subjects chose Love, followed by Truth and Freedom.

Using the Co-occurrence Explorer, we asked ATLAS.ti to show all codes that co-occur across all the transcripts. The result of this statistical process was a cross-tabulation of all codes. Data obtained through co-occurrence analysis between family indicators showed which dimensions, in respect to each Theme, were more developed inside the chosen events; the most co-occurrent codes and the respective events are: “love as family warmth” and “devoted love”; “value intended as love” and “moral value”; “power as authority” and “power as justice”; “freedom as natural condition” and “received freedom”; lastly, “subjective/objective truth” and “concealed truth.”

The peculiar co-occurrence of codes like “value to be passed on,” “value as respect,” “truth as guidance,” “value as responsibility,” “loss of values,” and “power as justice,” all attributable to the concept of ethical moral value, conducted us to introduce an additional Family called Ethic and Justice, which seemed to function as a background for themes like Value, Power, and Truth, as suggested by the peculiar recurrence of such gradients of meaning in terms of quotations. The codes suggested a semantic autonomy for this Family and we decided to consider it as such for the rest of our work, in order not to lose information from the data. The Table 3 shows the explicative indicators (codes), as well as examples of quotations.

Subsequently, we tried to connect the created codes to existing theoretical models described in literature. Specifically, we introduced Families to represent semantics to which we tried to associate codes assumed as representative for Love, Value, Power, Freedom, and Truth (Table 4).

**Table 4:** Code Families related to literature in the first Hermeneutic Unit (HU1).

Code Families	Codes	Quot.	Codes
		
	*n*	*n*	
Caring/Help (McAdams, 1996)	5	43	[Love as family value] [love as receiving material things] [incarnate love] [unconditional love] [mother’s love]
Certainty/Uncertainty (Sorrentino et al., 2008)	6	36	[lack of clarity] [lack of truth] [hidden truth] [truth as clarity] [truth as knowledge] [truth as discovery]
Responsibility (McAdams, 1996)	12	34	[Freedom as self-discipline] [power as discernment] [power as Justice] [value as liability] [value as respect] [value to be transmitted] [family value] [value of life] [value of things] [value as altruism] [moral value] [truth as respect]
Love/Friendship (McAdams, 1996)	5	30	[Love as trust] [love as passion, interest in something] [conjugal love] [love in friendship] [mutual love]
Freedom/Dependence Semantic (Ugazio, 2013)	6	27	[Necessity of freedom] [independence] [freedom as a conquest] [freedom as illusion] [freedom limited by boundaries] [freedom received]
Power Motive (McClelland, 1975)	10	25	[Achievement of power] [power management] [power as authority] [power as strength] [power as Justice] [power as threatening] [decision-making] [power at work] [power as abuse] [power related to money]
Self Mastery (McAdams, 1996)	11	23	[Self-control] [power management] [freedom management] [power as self-affirmation] [power as autonomy] [power as capacity] [power as discernment] [power as implementation] [decision-making] [power as possibility] [power as potential]
Exploration/Attachment (Ugazio, 2013)	8	19	[Love as passion, interest in something] [unconditional love] [freedom as a condition for being loved] [freedom as a natural condition] [freedom as the discovery and opening] [freedom of thought] [freedom of choice] [freedom of action]
Intimacy (McAdams, 1996)	8	16	[Love as trust] [exclusive love] [value as sharing] [value to be transmitted] [truth as dialogue] [truth as confidence] [truth as relation] [truth to be transmitted]
Freedom of thought	5	16	[Freedom of thought] [multiplicity of freedom] [hidden truth] [truth as knowledge] [truth to be transmitted]
Achievement Motive (McClelland, 1975)	8	12	[Power as ability] [power as fight] [power as implementation] [power as recognition] [power as will] [value as appreciation] [value as courage] [value as power]
Modus Amoris (Binswanger, 1942)	3	10	[Unconditional love] [indispensability of freedom] [freedom as a condition for being loved]
Status/Victory (McAdams, 1996)	6	10	[Power as recognition] [power as work] [recognition and appreciation of the value] [value as appreciation] [value as pride] [value as power] [criterion value]


According to the theory of McAdams and Guo (1980-1997), some codes could be included in the theme Love/Friendship and in the concept of Caring/Help, a concept relationship based on nurture, assistance and care (McAdams and Guo, 1980-1997). Codes for themes that reflect experiences of affection, connection and shared communication with someone else, like Truth, Value, and Love, are more closely related to the Family Intimacy (McAdams, 2001). Quotations from the theme Love seemed to be possibly linked, from phenomenological perspective, to Biswanger’s modus amoris: a way of being together inside love (Binswanger, 1942). Love as modus amoris is only possible when someone has the freedom to genuinely be that OneSelf able to merge into OurSelves.

Indicators for Power can be linked to different constructs according to semantic affinity: Achievement Motive, as defined by McClelland et al. (1976) in terms of personal fulfillment, success and empowerment; Status/Victory (McAdams and Guo, 1980-1997), corresponds to the recognition of personal values emerging from peer relationship; Power Motive, meant as the distinction, according to McClelland (1975), between personal and institutional (or social) power; Self Mastery, representing dominance and control, as well as power meant as ability to fulfill one’s own potential, Self-affirmation and Self-expression. Exploration/Attachment and Freedom/Dependence semantics (Ugazio, 2013) seem to implicate Freedom and Love themes in the dichotomy between being free and keeping emotionally relevant relationships with others: freedom and exploration are seen as values, while attachment and companionship are felt as the expression of the need for protection from a world perceived as threatening.

The semantic nucleus called Certainty/Uncertainty, representative of the theme Truth, unifies two distinct dimension of meaning that can be represented by the definition of Freedom as search for knowledge, clarity and objectivity/subjectivity. The construct Uncertainty Orientation (Sorrentino et al., 2008) was chosen because of its affinity between empirical data and the dimension including need for certainty and clarity maintenance as opposed to bearing ambiguity, curiosity and the desire to discover new or little known information.

### Studying the Quality and Occurrence of LTs in AAI

The second goal was studying the quality and occurrence of LTs in transcripts from the standard AAI interview, which composed the textual corpus for the second Hermeneutic Unit (HU2).

Grouping in Code Families was performed in order to clarify substantial meanings traced in the texts (the content of the Themes) in light of three different points of view that can be used to define the “sense” of a narration: sense as direction, movement and, inside a relationship, interpersonal arrangement; sense as frame, a limit or boundary in which experience unfolds; sense as flavor, intended as the ability to “feel” and attribute value to what is “felt.” Thus, the created categories include each Theme within: the main interpersonal and intrapsychic positions (direction); the description of experiences in function of self-boundaries (frame); the positive or negative valence attributed to narrated events (flavor).

Three main relational and intrapsychic movements were identified in the transcripts: from oneself to someone else (position of giving), from someone else to oneself (position of receiving), from oneself to its own self (position of self-reflection). Such partitioning criteria was applied in a balanced and symmetrical manner, in order to outline a complete theoretical model. On a descriptive level, the most rooted codes are homogeneously distributed throughout each Theme; however, the relational movement with the highest frequency was receiving. Table 5 shows the indicators of Code Families of the themes in relations to these dimensions. Moreover, AAI standard protocol includes a specific item designed to explore the dimensions of loss and grief. Since many codes emerged about grief, we decided to introduce Code Families ad-hoc, in order to consider each theme in function of loss and grief as a boundary conditions. We considered such dimension as a boundary domain, which pushes the narrator to come to terms with the absolute limit and borrow his own narrative style about the Themes from it.

**Table 5:** Code Families in the second Hermeneutic Unit (HU2).

Code Families	Codes	Quot.	Positive/negative description
		
	*n*	*n*	
Position of receiving love	8	89	Experiences of: affection and proximity, received and felt, in terms of physical warmth, devotion, acknowledgment of uniqueness and attention to the person’s needs/lack of affection and proximity from others, in terms of neglectfulness and rejection of affection requests, carried out by the subject.
Position of giving love	5	164	Experiences of: expression, demonstration, granting of affection and proximity to the others, in terms of physical warmth/active forms of rejection and opposition toward the expression of affection and proximity.
Position of being lovable	1	3	Experiences connected to: being and feeling lovable, charming and pleasant, able to receive attention and physical warmth/a sense of intrinsic inability to be seen as lovable, charming, worthy of affection.
Position of receiving value	4	144	Experiences connected to: having been appreciated according to an external criterion or in comparison to a model/having been appreciated because of intrinsic qualities, without referencing an external criterion/having been devalued and negatively judged, with regard to one’s own qualities.
Position of giving value	2	24	Experiences of: actively attributing a qualification to someone based on an external criterion/qualification of others in explicit relation to an external model.
Position of being valuable	2	26	Experiences connected to: self-acknowledgment of qualities and merits/sense of a lack of personal qualities and merits/achievement of esteem, inner talents.
Position of being subjected to someone’s power	4	131	Experiences connected to: having been controlled, bossed around, threatened, by means of actions that require being subjugated/having been raised with a dialogue-based discipline.
Position of exerting power			Experiences of: active control and imposition on the others, threats and situation implying the superiority of the subject toward the others/lack or impossibility to exert active control, imposition on the others.
Position of personal potential	1	6	Experiences connected to: the ability to be oneself and the sense of self-control/lack of the ability to be oneself/self-control, self-discipline.
Position of receiving freedom	2	102	Experiences connected to: having been given space for self-expression, action, thought and self-determination/having been subjected to obligations, constraints, restrictions of one’s own space.
Position of giving freedom	–	–	Experiences connected to: actions driven by claims of one’s own space for self-determination and expression/granting space for the others’ expression/limitation, through obligations, of the others’ ability to behave and express themselves.
Position of being free	4	60	Experiences connected to: the condition of feeling loosen, untied, in terms of independence and self-sufficiency.
Position of receiving truth	–	–	Experiences connected to: having acquired, learned, discovered from someone else a reality, a fact, a meaning, a secret/the lack of disclosure of facts, by concealing, omission, deceit or lie.
Position of giving truth	7	116	Experiences connected to: passing down or communicating to others information about the reality, facts, meanings, thought disclosure, confidence/lack of communication of facts, meanings, secrets.
Position of the sense of self	5	100	Experiences connected to: the feeling of being genuine and truthful/the clarification and/or discovery, acquisition, interpretation, awareness of a meaning related to one’s own personal story/the search for authenticity, meaning, through question, reflections.
Position of receiving justice	3	3	Experiences connected to: having received an equal, regular, rightful, treatment, the respect of one’s own rights/having received an unjust, dishonest treatment, with violations of one’s own rights.
Position of giving justice	3	5	Experiences connected to: having granted to others an equal, regular, rightful, treatment and having acknowledged and respected others’ rights/having exerted an unjust and dishonest treatment, with violations of others’ rights.
Position of ethic and morality	3	8	Experiences connected to: the feeling and acknowledgment of being rightful, fair, respectful of ethical and moral standards.
Affection and Loss	4	42	Description of the response to a loss as: grief, lack, deprivation of affection and proximity to the one who passed away/continuity and stability of affection and emotional connection to the one who passed away.
Value and Loss	2	26	Description of the response to a loss as: inner growth, acquire and strengthening of personal attributes, acknowledged to oneself as well as others/annihilation, reduction, and loss of personal attributes.
Freedom and Loss	2	14	Description of the response to a loss or the idea of death as: liberation and emancipation from living constraints placed by oneself or others/absolute limitation and insurmountable frontier.
Power and Loss	2	7	Description of the response to a loss or the idea of death as: an overwhelming event, an explicit or implicit threat that imposes itself ultimately controlling one’s life and/or put an end to all/annihilation of both one’s own potential and the possibility to be oneself/a chance for restoring personal attributes.
Truth and Loss	2	2	Description of the response to a loss or the idea of death as: acknowledgment of the sense of the event/search or discover of new contents and meanings.
Justice and Loss	2	–	Description of the response to a loss or the idea of death as: fairness, righteousness, logical consequence, validity of the event/iniquity of the event/liberation, compensation for previous situations.


We grouped some codes in Code Families concerning the subject’s position relative to Self-boundaries. The personal boundaries constitute borders or limits that delimit the space that surrounds the individual, within which he can feel himself and to be safe (Bell et al., 1996). We can distinguish a sense of somatic boundaries (Ogden et al., 2006), a sense of inter-personal boundaries defining the distance between self and others, a sense of the self-boundaries in terms of self-representations. The concept of boundary emphasizes the idea that its dimensions will vary according to the specific situation and to internal states. This semantic category was developed with the aim of understanding those codes that, regardless of the topic to which they belonged, indicated in the narration the positioning of the others outside, inside or near the personal boundaries. In the transcripts this concept emerged in relation to the Themes in terms of proper distance, invasion or extreme detachment from significant figures (Table 6). Indeed, as suggested by Bar-Haim (2002), early strategies employed in regulating the caregiver’s proximity are progressively represented inside the Internal Working Model, subsequently influencing both the regulation of one’s own personal space and the shape of one’s own boundaries concerning interpersonal relationships.

**Table 6:** Code Families associated to the subject’s position relative to Self-boundaries.

Code Families	Codes	Quot.	Description and examples of codes
		
	*n*	*n*	
Position relative to Self-boundaries: External	8	135	The relationship with the others is narrated in terms of detachment, reciprocal independence, lack of affection, self-enhancement through external criteria. [Lack of received affection, refusal of demonstration of and requests for affection]
Position relative to Self-boundaries: internal	10	205	The relationship with the others is narrated in terms of intrusiveness of one’s own boundaries, impositions suffered by the other, failure to recognize one’s own needs due to too much involvement in the others’ needs, role reversals in the attachment bond. [Use of a personal sense of morality] [Display of an intrusive and harassing affection] [Having been subjected to impositions]
Position relative to Self-boundaries: proximity	5	159	The relationship with the others is narrated in terms of mutual respect of personal boundaries, respectful recognition of personal needs, trust in bringing the other closer to one’s personal space. [Understanding and acknowledging a personal truth] [Display of affection and proximity in terms of dedicated and appreciative love]


### Preliminary Analysis of the Possible Association Between LTs and State of Mind in Regards to the Attachment

Our goal was not to investigate a possible association between LTs and Internal Working Models, rather to operationalize the definitions. Nevertheless, it was possible to preliminarily explore the distribution of the thematic indicators obtained from the AAI groups. Transcripts were grouped together by state of mind. We chose to use the three-way distribution of state of mind, instead of the four-way one. For the purposes of this study focusing on content analysis, we excluded U transcripts because the placement in these groups typically is based on a few phrases or a single linguistic passage, while the state of mind according to the three-way distribution represents the general strategy of the subject. The general strategy is conceptually closer to the LTs. The absolute number and percentage of quotations for each indicator (Code Family) were calculated in F, E, and Ds (Table 7). AAI groups do not significantly differ (Kruskal–Wallis = 6.06; d.f. = 3; *p* = 0.1), nevertheless it is possible to draw some qualitative conclusions. As indicated by bolded values, the indicators “Receiving value” and “Giving love” were more frequent in transcripts from the Ds group, compared to the others (15,5 and 16,1%, respectively, *n* = 316). In the E group (*n* = 300), 16% were coded with the indicator “Being subjected to someone’s power,” which is the most frequent indicator in this group, followed by “Receiving value” (12,7%). The most frequent indicators in the F group (*n* = 420) were “Giving love” (19%) and “Giving truth” (13,6%).

**Table 7:** Frequency distribution of indirect indicators in the AAI Groups.

Code Families	DS		E		F	
			
	*n* *quot.*	*%*	*n* *quot.*	*%*	*n* *quot.*	*%*
Position of being free	15	4,7	24	8,0	21	5,0
Position of giving love	49	**15,5**	33	11,0	80	**19,0**
Position of giving value	7	2,2	9	3,0	8	1,9
Position of giving truth	42	13,3	16	5,3	57	**13,6**
Position of receiving love	15	4,7	29	9,7	37	8,8
Position of receiving freedom	28	8,9	32	10,7	41	9,8
Position of receiving value	51	**16,1**	38	**12,7**	54	12,9
Position of being subjected to someone’s power	35	11,1	48	**16,0**	24	5,7
Position of the sense of self	30	9,5	26	8,7	43	10,2
Position of being lovable	0	0,0	0	0,0	3	0,7
Position of exerting power	0	0,0	1	0,3	2	0,5
Position of exerting justice	2	0,6	1	0,3	2	0,5
Position of being valuable	6	1,9	15	5,0	5	1,2
Position of receiving justice	0	0,0	0	0,0	2	0,5
Position of ethic and morality	5	1,6	1	0,3	2	0,5
Position of personal potential	1	0,3	3	1,0	2	0,5
Affection and Loss	8	2,5	6	2,0	28	6,7
Freedom and Loss	7	2,2	4	1,3	3	0,7
Power and Loss	5	1,6	1	0,3	1	0,2
Value and Loss	9	2,8	13	4,3	4	7,4
Truth and Loss	1	0,3	0	0,0	1	0,2
Total	**316**	**100**	**300**	**100**	**420**	**100**


Concerning different positions in relation to self-boundaries (Table 8), the secure group (F) chose positioning in proximity more often than the insecure groups (Ds and E), which is related to relationships based on consideration and respect of one’s own personal space as indicated by bolded values.

**Table 8:** Frequency distribution of indicators of the position relative to Self-boundaries in the AAI groups.

Code Families	DS		E		F	
			
	*n* *quot.*	*%*	*n* *quot.*	*%*	*n* *quot.*	*%*
Position relative to Self-boundaries: external	45	29,8	48	27,9	42	23,9
Position relative to Self-boundaries: internal	54	**35,8**	94	**54,7**	57	32,4
Position relative to Self-boundaries: proximity	52	34,4	30	17,4	77	**43,8**
Total	**151**	**100**	**172**	**100**	**176**	**100**


## Discussion

The main goals of the present study concerned: the systematization of the theoretical model related to the constructs of the LTs (throughout the identification of direct and indirect indicators); matching the emergent meanings with theoretical models available in literature; the preliminary exploration of the association between LTs and State of Mind in regards to the attachment.

Regarding the aim related to the identification of direct indicators of the LTs, our results showed which shades of meaning are attributed by the subjects to each Theme upon direct request. Lexical analysis resulted in the creation of specific thematic dictionaries, comprising keywords for detecting the Themes.

Constructs were broken down into various indicators and indicators were grouped to achieve a higher level of theoretical abstraction. Groups constituted a semantic network. Notably, strong correlations were found between Themes, like Power and Freedom or Power and Value, as indicated by co-occurrence of specific thematic dimensions in the same portions of transcripts. The investigation of such connections enabled us to match the emerged semantic topics with constructs available in literature.

The second aim was investigating the indirect indicators of the LTs in the attachment stories (gained through the standard AAI protocol), in order to enrich the autobiographical narrations with semantic dimensions, uncovering the interpersonal relational movements.

Love, described as influence, emerges mostly in controlling and oppressing situations, where the main way of obtaining affection is represented by the obligation to respect rules and impositions. Obedience and respect are valued as currency for love.

Value emerges in both the Hermeneutic Units (direct and indirect indicators) as moral sense. Subjects understand the concept of value as part of an abstract moral system, as an inspiring principle, as they were probably raised to do; moreover, value is intended as a peculiar passion, a distinctive trait of personality. Giving Value, in contrast to acknowledging it, seem to concern the confrontation with the esteemed loved one; at time it is meant as efficiency, regarding pragmatical features experienced in infancy, starting from the valorization of discipline and rigor. Personal Value is intended in two ways: the ability to “tame” the other, influencing his/her development and the acquired ability to show reverential respect for the authority.

Power is mostly intended as imposition, whereas the attachment relationship with the caregivers was characterized by submission to rigid rules. It is associated with manipulation, a sense of constraint connected to limited freedom; when considered in light of an asymmetric attachment relation, it might also represent a point or reference for providing stability during infancy. It is connected to situations where the interpersonal motivational system of competition for resources and forming social ranks (Liotti, 2001; Liotti and Monticelli, 2008) emerges: starting from a conflictual relationship, facilitated by a suffocating sensation of being controlled and a judgmental context, an asymmetric balance of power arises, becoming the framework of reference for the attachment bond.

Freedom seems to characterize experiences of controlling and imposing situations: the strenuous achievement of material and intellectual freedom; a prerequisite for experiencing. The sensation of Freedom, meant as the ability to experience the world, as movement and change, originates from the awareness of a steady point, a reference to return to, as way of finding stability.

Truth emerges as the search for sense, rational answers and explanations inaccessible during infancy. It develops as individual or parent-guided search, which requires courage and strong will to be pursued; it cannot be given, it must be discovered and, once found, accepted. In its negative connotation of deceit, omission or dissimulation, Truth represents the only possibility of achieving freedom inside a relationship; in other narrations it represents the mask used to conceal oneself, in order to avoid suffering that might originate from having to deal with deeper issues.

Justice characterizes those sections of the interview associated with reflecting on the motivations behind the caregiver’s behavior: it can be intended in terms of receiving a fair treatment, also in lights of one’s own achievement, or as a form of recrimination for wrong doings that still disrupt the relation’s representations.

Results from the preliminary exploration of association between LTs and the attachment’s states of mind suggest a link between dismissing subjects’ narrations (Ds) and the theme Value, when considered in the dimension of receiving. This result seems to be in line with an idealizing narrative style, aimed at emphasizing one’s own strength and resilience as positive outcome of distressing situations (Jacobvitz et al., 2002). Narrations characterized by a preoccupied/entangled style (E) are mostly centered on the theme Power, in terms of “Being subjected to someone’s power” within the attachment relationships. This result seems to be in line with the high levels of disapproving anger and complaints toward the caregivers typical of this state of mind: these transcripts are often characterized by unbalanced, excessive blaming of either parents or self. Moreover, preoccupied adults often report higher levels of psychological control from their parents and an unproductive overengagement with parents in arguments that ultimately undermine their autonomy (Allen and Hauser, 1996; Hesse, 2016). The high occurrence of the Power theme may be related to the experiences in which emotionally rejecting or hostile caregivers.

In general, individuals with an insecure state of mind tend not to believe they deserve others’ appreciation about their value and to feel needy or inadequate.

Transcripts that showed a high level of coherence (F) seem to include each Theme in a more or less balanced manner, with a slight prevalence of the theme Love, meant as warmth and unconditional reciprocity: indeed, such state of mind reflects an intrinsic ability to explore one’s own thoughts and feelings in relation to the examined thematic, providing a well aware description of factual reality and attributed meanings (Main and Goldwyn, 1984; George et al., 1985; Hesse, 2016). For the future analysis that may include the U transcripts we expect a particular association between the disorganization typical of such transcripts, when involving the narration of unresolved traumas or loss, and the themes Truth (dimension of the sense of self) and Value (dimension of receiving), in terms of disorientation in the personal and existential sense of self (Hesse and van IJzendoorn, 1999; Hesse, 2016; Lyons-Ruth and Jacobvitz, 2016).

In relation to the dimension of Positioning in respect to one’s own boundaries, the secure subjects tend to interpret it as proximity. We believe that such attitude might indicate a representation of relationships in terms of adequate. In fact, free/autonomous adults report more proximity and sensitivity than dismissing ones, that tend to suppress and deny worries, affection and intimacy. The insecure subjects’ narrations focus on the intrusion of one’s own personal space or on the tendency to impose an increased distance between interpersonal relations. Preoccupied individuals consistent with their strong need for approval and their hyperactivation of the attachment system report persistent worries about attachment figures’ availability that lead them to frequently seek the proximity and to blur the distinction between another person’s boundaries. In this subjects there is the juxtaposition between the desire for intimacy and proximity and the concern about not receiving suitable care (Waldinger et al., 2003).

Distance between individuals, which in turn provides a better understanding and awareness of one’s own boundaries, as well as a flexible and efficient balancing (Bar-Haim et al., 2002).

## Conclusion

The general idea behind the present work was to elucidate the LTs (as formulated by Veglia, 1999, 2013; Veglia and Di Fini, 2017), their coherent shared semantic organization, recurrent in autobiographical recollections obtained through the Adult Attachment Interview. We aimed at complementing the analysis of the narration’s formal coherence, the representational processes and the ways of constructing the experience, with a formal reflection upon the unfolding of six LTs within the boundaries of the motivational attachment system’s activity. Qualitative analysis of AAI transcripts leaded to a systematization of the theoretical hypothesis, able to appropriately account both for the content and themes found in life stories. On the clinical level, the reconstruction of life stories first and the search for meaning inside them second, are at the core of the therapeutic activity. The outlined thematic indicators might be useful as warning devices for identifying dysfunctional critical schemes and cognition.

The subjects who encounter difficulties in reorganizing their own experiences and harmonizing the Themes in relation to a personal code of coherence, might be more vulnerable to the impact of devastating emotions, possibly evolving into psychopathologies of the sense and significance.

Our goals were to design the operational definitions of the LTs, the analysis of the semantic networks, uncovering the interlinks between meanings and, finally, the preliminary observation of the prominent Themes in the AAI groups. The results we obtained represent a frame of reference for hypotheses that will need to be verified in future studies. Future directions of the study should include an analysis of the relationship between scores on AAI scales and LTs.

This study’s strengths come from its attention to the link between the attachment (the Internal Working Models), the Life Themes and their operational definition. It aims to propose an innovative approach in the study of language and representations applied to the narratives of adult attachment experiences. In terms of clinical applications, the analysis of therapeutic progress is important not only in terms of narrative coherence and reflective ability, but also as a measure of balanced development between critical themes.

An interpretative and explicative model of the patient’s critical Themes, associated to past attachment experiences, able to complement information regarding different states of mind, both thematically and linguistically, might open the way for a deepened understanding of the patient’s schemes for building knowledge. It is important to emphasize that our results suffer from the methodological limitations arising from small samples. Generalization of our results is limited by sampling from a non-clinical population and by the subjects’ age (between 22 and 50 years old) and education (medium–high). Increasing the sample size will be of primary importance for future studies, in order to better investigate occurrence and quality of thematic indicators, ultimately being able to completely formalize a systematic theoretical model, capable of better orienting both the researcher and the clinician in understanding the patient’s narrative contents.

## Author Contributions

FV provided the theoretical insights and clinical foundations about the hypothesis of Life Themes. GDF contributed to the conception and development of the manuscript. GDF and FV designed the project, conducted the studies, oversaw all aspects of analysis and interpretation, and have made a substantial, direct and intellectual contribution to the work, and approved it for publication.

## Conflict of Interest Statement

The authors declare that the research was conducted in the absence of any commercial or financial relationships that could be construed as a potential conflict of interest. The handling Editor declared a shared affiliation, though no other collaboration with the authors GDF and FV at the time of review.
